# Glucose Metabolic Abnormality: A Crosstalk between Depression and Alzheimer’s Disease

**DOI:** 10.2174/011570159X343281240912190309

**Published:** 2024-09-24

**Authors:** Shaobin Yang, Yanhong Li, Qi Tang, Yimeng Zhang, Tingji Shao

**Affiliations:** 1 College of Life Sciences, Northwest Normal University, Lanzhou, Gansu, 730070, China;; 2 Department of Pharmacy, Gansu Provincial People’s Hospital, Lanzhou, Gansu, 730000, China

**Keywords:** Depression, glucose metabolism, Alzheimer’s disease, insulin signaling, neuron, therapeutic strategy

## Abstract

Depression and Alzheimer’s disease (AD) are two prevalent and debilitating conditions that significantly impact millions of people worldwide. Depressive disorders are characterized by persistent feelings of sadness, loss of interest, and impaired cognitive function. AD is a progressive neurodegenerative disorder that is accompanied by cognitive decline, memory loss, and behavioral changes. To date, the pathogenesis of AD and depression has not yet been fully explained. Recent studies have provided insights into the intricate relationship between these two disorders by emphasizing the role of glucose metabolic abnormalities as a potential link. This review explores the bidirectional association between depression and AD, focusing on common pathophysiological mechanisms involving glucose metabolism, such as hypothalamic-pituitary-adrenal (HPA) axis dysregulation, insulin resistance, glucose transporters, and oxidative stress. Understanding the crosstalk between glucose metabolic abnormalities, depression, and AD will open new avenues for therapeutic interventions. Finally, improving glucose metabolism through lifestyle modifications, pharmaceutical interventions or novel therapeutic approaches could provide a promising therapeutic strategy for managing both conditions simultaneously.

## 1 INTRODUCTION

Alzheimer’s disease (AD) is the most common form of dementia [1]. It is characterized by the progressive deterioration of memory and cognitive function, along with the development of primary progressive aphasia, self-dissociation, and behavioral abnormalities [2, 3]. AD affects over 50 million people worldwide, and the number is expected to triple over the next four decades [4]. So far, there has been no effective treatment to decrease progression due to in adequate understanding. Furthermore, AD is primarily associated with two irreversible pathologies, a cluster of neurofibrillary tangles and amyloid plaque conformation, which are accompanied by synaptic loss and neuronal death [5, 6]. Metabolic dysfunction including impaired glucose metabolism, is another important feature of AD, and, patients and transgenic AD model mice display a reduced brain glucose metabolism [7]. Recent studies have shown that diabetes mellitus type 2 (T2DM) and AD share common pathogenesis features linked to glucose metabolic abnormality, insulin resistance, oxidative stress, and inflammation [8-13].

Depression is a common mental health disorder marked by persistent feelings of sadness, hopelessness, and lack of interest [14]. Over 264 million people worldwide suffer from depression, which is a leading cause of disability [15]. Recent research has suggested a potential bidirectional relationship between depression and glucose metabolism [16]. Depression may disrupt and glucose metabolism, lead to metabolic disorders such as diabetes [17]. T2D is associated with an increased risk of depression [18]. Hyperglycemia, oxidative stress, and neuroinflammation induced by T2D have been demonstrated to induce depression-like behavior in diabetic mice [18, 19]. On the other hand, metabolic disorders can worsen depressive symptoms, resulting in a detrimental cycle [20].

However, the exact causes of AD and depression are not yet fully understood, recent studies have highlighted glucose metabolic abnormalities as potential links between these disorders [21]. Usually, both disorders are viewed as separate conditions; however, emerging evidence suggests a bidirectional relationship between depression and AD [22]. Further, understanding the role of glucose metabolism may unravel both disorders’ complexity and identify common treatment pathways, providing a promising avenue for future investigations. Cells require glucose for energy, and their levels should be controlled in the body and cells. Nonetheless, impaired glucose uptake affects mitochondrial ATP production and can cause AD and depression [7, 23, 24]. Additionally, depression and AD are associated with dysregulation of the hypothalamic-pituitary-adrenal (HPA) pathway, which regulates immune function and brain glucose metabolism [25-27]. Thus, it is necessary to explore new treatment strategies for AD and depression based on their pathogenesis. Some drugs target glucose metabolism disorders and improve brain metabolism and regeneration by reducing the neuropathology associated with AD and depression. This review provides molecular insights into the common pathogenic mechanisms that link these disorders.

## 2 PATHOLOGICAL FEATURES OF ALZHEIMER’S DISEASE AND DEPRESSION

**Fig. (1):**
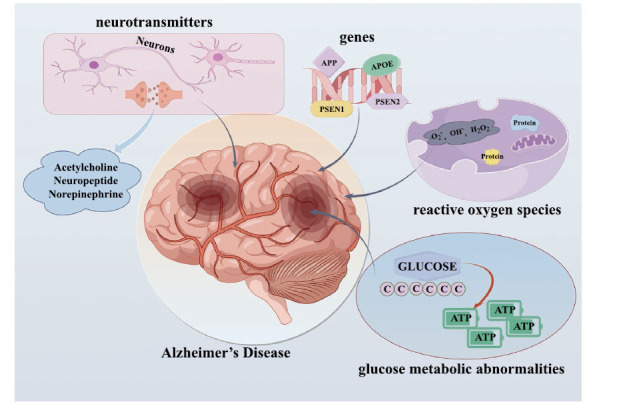
Multifactorial basis of the features of Alzheimer’s disease. Hypotheses regarding genetics, neurotransmitters, glucose metabolic abnormalities, reactive oxygen species (ROS), and inflammation have been promoted to explain this multifactorial disorder. Aβ production increases with *APP*, APOE, *PSEN1* and *PSEN2* gene mutations, leading to impairment of neuronal activities. Neurotransmitters including acetylcholine, neuropeptide, and norepinephrine impair synaptic functions. ROS activates inflammatory pathways and disrupts synaptic function in AD. Tau hyperphosphorylation and glucose metabolic abnormalities could cause AD.

Dr. Alois Alzheimer first reported the neuropathological characteristics of AD in 1907 [28]. Extracellular neuritic plaques containing misfolded amyloid-beta (Aβ) proteins and intraneuronal neurofibrillary tangles (NFTs), caused by the accumulation of hyperphosphorylated tau proteins, are the main features of AD [29-31]. Several factors, such as genetics, neurotransmitters, glucose metabolic abnormalities, reactive oxygen species (ROS), and inflammation, play important roles in developing this multifactorial disorder (Fig. **1**) [32-37]. Generally, depression is defined by constant feelings of sadness and a lack of interest in activities that are usually enjoyed, and symptoms include energy loss, change in appetite, anxiety, or feelings of worthlessness [38]. However, severe depression can lead to self-harm or suicide [39]. Although the fundamental mechanisms underlying depression remain unknown, alterations in neurotransmitter systems, neuroinflammation, and neuroendocrine dysregulation have all been implicated in this disorder [40-44].

### 2.1 Tau in Alzheimer’s Disease

Accumulation of phosphorylated Tau is the main pathological feature of AD [45]. Tau is essential for maintaining neuronal structure and function, regulating the assembly and stabilization of microtubules, and nutrient transport within nerve cells [46]. However, abnormal Tau protein undergoes many alterations that lead to the formation of neuronal tangles, impaired nutrient transport, and, ultimately, cell death in AD [47]. Tau is a component of the normal microtubule network and is essential for axonal transport and communication between axonal compartments and somatodendritic regions [48]. However, the accumulation of Tau tangles is thought to contribute significantly to the cognitive decline observed in AD [49]. The hyperphosphorylation of Tau proteins forms these tangles [50]. This hyperphosphorylation leads to the detachment of Tau proteins from microtubules, consequently clustering together and forming neurofibrillary tangles [51]. These tangles block the transport system, eventually leading to neuronal death [52].

Interestingly, research indicates that the accumulation of Tau tangles in the brain is more closely associated with cognitive decline in AD than beta-amyloid plaques, suggesting that Tau pathology is more closely related to neurodegeneration [53]. The progression of Tau tangles throughout different brain regions is related to the development of clinical symptoms in AD. Further, recent studies have suggested that Tau protein may contribute to the spread of AD within the brain [54]. It is hypothesized that abnormal Tau proteins can be transferred from one neuron to another, triggering recipient neurons to develop Tau tangles [55]. As Tau pathology spreads from the initial sites to other areas of the brain, this mechanism could explain the progressive nature of AD [56]. The significance of Tau in AD has spurred increased interest in developing novel therapeutic approaches targeting Tau. These therapeutics are designed to inhibit Tau phosphorylation, prevent its aggregation, and potentially modify the course of AD.

### 2.2 Genetics of Depression

**Fig. (2):**
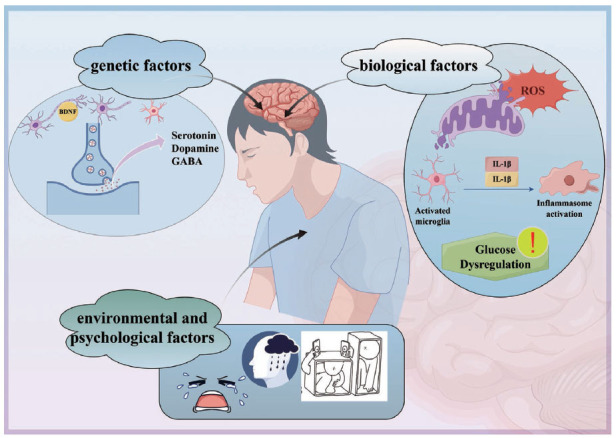
Depression is a complex disorder resulting from a combination of genetic, biological, environmental, and psychological factors. Serotonin, Dopamine and GABA gene mutations, leading to impairment of neuronal activities. Biological factors including activated microglia, inflammasome activation, ROS activates inflammatory pathways and glucose metabolic abnormalities leads to depression. Comorbidities, such as environmental, psychological factors could also cause depression.

Depression is a complex disorder resulting from a combination of genetic, biological, environmental, and psychological factors (Fig. **2**) [40]. Chemical imbalance is a common factor contributing to depression in the brain [57]. Neurotransmitters such as serotonin and dopamine are critical in regulating mood and emotions [58].

Studies have demonstrated that a positive family history is one of the most important risk factors for developing depressive disorder (40-50%) [59]. One of the key genes associated with depression is the serotonin transporter [60]. Serotonin regulates mood, memory, sleep, and body temperature, and low serotonin levels are associated with depression [61]. Selective serotonin reuptake inhibitors (SSRIs) are commonly prescribed to increase the serotonin levels in the brain [62]. SSRIs elevate the concentration of 5-hydroxy-tryptamine (5-HT) in the synaptic spaces of serotoninergic neurons, leading to a therapeutic impact on depression [63]. Various 5-HT receptors, such as 5-HT1A and 5-HT1B, modulate depression and mood-related behaviors [64]. Behavioral depression is closely related to 5HT1A deficiency and disruption of the cAMP/PKA/CREB pathway in the hippocampus [65]. Depression caused by chronic stress is characterized by an increase in serum corticosterone (CORT), a decrease in serotonin, and an elevated level of IFN-γ and TNF-α in the frontal cortex of rats [66]. Another gene involved in depression is brain-derived neurotrophic factor (BDNF), which plays a vital role in neuronal growth and survival, and its decreased levels are linked to depression [67]. Additionally, BDNF gene variations have been linked to an increased risk of developing depression, as they can affect the brain’s ability to adapt and respond to stress associated with 5-HT [68]. 5-HT receptor stimulation produces rapid and sustained antidepressant-like effects by activating the BDNF-mTOR signaling pathway in the medial prefrontal cortex [69]. In addition to these genes, some studies have identified several other genetic variations associated with an increased risk of developing depression. Opioid receptors are widely distributed in the frontal cortex and limbic regions, such as the amygdala and hippocampus, and are involved in mood and pain [70]. Dopamine plays a major role in regulating rewards, mood enhancement, and overcoming depression and aversion [71]. Gamma-aminobutyric acid (GABA), an inhibitory neurotransmitter, affects approximately one-third of neurons in the brain and has been shown to be effective in treating depression and mania [72]. In depression, an increase in glutamate and glutamine levels is associated with a decrease in GABA levels, and glutamate stimulation toxicity causes GABA dysfunction [73].

### 2.3 Glucose Metabolism and Alzheimer’s Disease

The human brain utilizes 20% of the glucose; it requires a great deal of energy mainly to uphold the synaptic activity; 95% of glucose is employed in the generation of ATP [74]. This is the reason that alterations in glucose metabolism led to harm to cell regulation, as reduced ATP can impact proper synaptic function. A large part of the process is independent of insulin regulation. Nevertheless, there are insulin receptors in diverse brain regions, influencing such processes as memory, cognition, and the regulation of energy metabolism [75]. Glucose homeostasis is critical for inhibiting AD, as reduced glucose levels in the CSF and brain can increase synaptic inactivity and the risk of AD [76]. According to previous studies, individuals with diabetes have a higher risk of developing AD than those without [77]. Also, AD is characterized by impaired glucose metabolism in the brain, often referred to as “type 3 diabetes [78]”. Further, AD patients observed reduced glucose metabolism and utilization in affected brain regions using the positron emission tomography (PET), such as the hippocampus and frontal cortex, which leads to cognitive decline and neurodegeneration [79]. Several expressions of glucose transporters were reduced in the brain of AD model mice [80]. Inside cells, glucose is metabolized either through the pentose phosphate pathway (PPP) or by means of glycolysis [81]. Hexokinase (HK) is the first-step catalytic enzyme, and phosphofructokinase (PFK) is another rate-limiting enzyme in glycolysis, while glucose-6-phosphate dehydrogenase (G6PD), which is regarded as one of the most crucial enzymes for controlling the rate-limiting step of the PPP, their activities were discovered to be decreased in the cortex and hippocampus [82]. In addition, the effects of insulin on the brain, which can lead to mitochondrial dysfunction, oxidative stress, and inflammation, are key factors in glucose dysregulation [35, 83]. These glucose metabolic abnormalities in AD are thought to be activated by amyloid-beta-induced insulin resistance, mitochondrial dysfunction, and oxidative stress [84]. Moreover, these processes cause neuronal damage and cognitive decline by disrupting energy production, impairing synaptic function, and promoting neuroinflammation [85]. Thus, it is possible that although brain glucose metabolism impairment can relate to AD, the mechanism is still inconclusive [86]. High blood glucose levels can also negatively affect the brain because excess glucose can form toxic protein aggregates known as amyloid plaques, which involves a glucose analog to evaluate glucose metabolism in the brain, has been proposed as a potential biomarker for AD [87].

#### 2.3.1 Mechanisms Linking Glucose Metabolism and AD

**Fig. (3):**
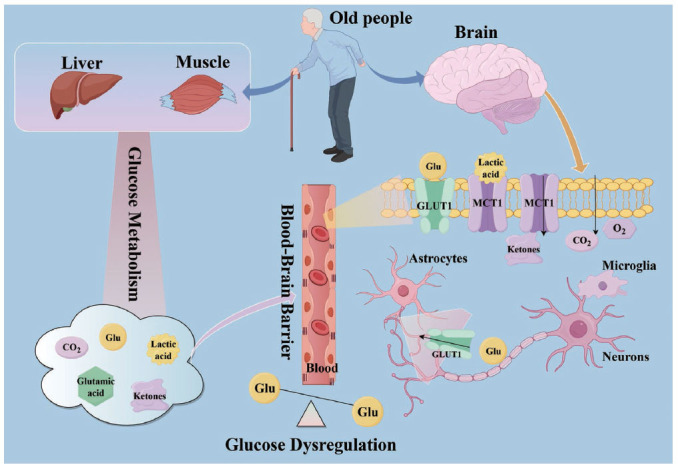
Specific mechanisms by which aging improves AD glucose metabolism. Insulin regulates glucose metabolism in liver, muscle, and brain. Glucose across the blood brain barrier and it is transported into astrocytes *via* glucose transporter (GLUT) 1, whereas the major transporters of glucose in neurons and microglia are GLUT3 and GLUT5.

Insulin is a hormone that regulates glucose metabolism. The peripheral regions of the body need insulin to activate the signaling that enables the translocation of glucose transporters, facilitating the entry of glucose into the cell [88]. For instance, the insulin signaling pathway governs the translocation of GLUT4 to the plasma membrane [89]. It involves the binding of insulin to its receptor (a tyrosine kinase), which will phosphorylate the substrate proteins of the insulin receptor (IRS1/2) and recruit adaptors to the PM, like phosphatidylinositol 3-kinase (PI3K). PI3K raises phosphatidylinositol 3,4,5-trisphosphate (PIP3) at the PM, resulting in the activation of protein kinase B [89]. In the central nervous system (CNS), insulin must cross the blood-brain barrier (BBB) and bind to its receptor, and the conformational alteration of which leads to the enzymatic activity of tyrosine kinase and the autophosphorylation of the receptor [90]. In addition to glucose homeostasis, it is important for maintaining brain function, including neuronal function, promoting the creation of new memories, and regulating neurotransmitter levels in the brain (Fig. **3**) [91]. However, insulin resistance associated with type 2 diabetes can cause the brain to resist the effects of insulin, impair cognitive function, and increase the risk of developing AD [92]. According to these studies, insulin resistance impairs glucose uptake in the brain and promotes the accumulation of amyloid-beta plaques, resulting in energy deficits and neuronal dysfunction [93]. Also, it has been found that insulin resistance and diabetes may contribute to the development of AD through other mechanisms, such as mitochondrial dysfunction, oxidative stress, and neuroinflammation, along with glucose dysregulation in the brain [94]. Further, insulin resistance (IR) increases the production of inflammatory molecules in the body and promotes the progression of neurodegeneration. IRs are extensively expressed in diverse brain regions, such as the hypothalamus, hippocampus, cerebral cortex, striatum, cerebellum, choroid plexus, and olfactory bulb. IR along with insulin-like growth factor 1 (IGF1) resistance (IGF1R) has been stated to be present in neurons from the human postmortem hippocampi of AD subjects, entailing flaws in the signaling cascades after insulin receptor activation along with resistance (IGF1R) has been stated to be present in neurons from the human postmortem hippocampi of AD subjects, involving defects in the signaling cascades after insulin receptor activation [95]. Insulin signaling is vital for neuronal growth, as it regulates neurotransmission and synaptic plasticity *via* the Mitogen-Activated Protein Kinase (MAPK) and Akt signaling pathways [92, 96]. Dysregulation of these pathways is essential for producing Aβ, which increases the activation of glycogen synthase kinase 3β (GSK3β) to enhance Tau phosphorylation [97]. Impaired insulin signaling is related to the accumulation of neurotoxic Aβ and hyperphosphorylated Tau through decreased PI3K/AKT signaling and enhanced activation of GSK3β [98]. Additionally, it was demonstrated that IR/IGF1R in AD brains involves decreased receptor binding and IRS/PI3K/PKB pathway activation, along with reduced anti-apoptosis-related mechanisms of insulin/IGF1 [99]. Conversely, Aβ can disrupt insulin signaling and downregulate the PI3K/Akt pathways [100]. Furthermore, there is increased expression of brain insulin resistance markers, IRS-phosphorylated at serine 616 (IRS-1 p^S616^) and IRS-1 p^S636/639^, in the hippocampus and cerebellar cortex of AD patients without T2DM [101].

Furthermore, Yang *et al*. reported that the cortical and hippocampal PDK1 phosphorylation at Ser241 in AD mice increases with aging, which subsequently increases Akt phosphorylation at Thr308 and Ser473, which significantly increases within the hippocampus and cortex of 3xTg-AD mice [102]. Increased phosphorylation of PRAS40 at Thr246 leads to Akt hyperactivity in AD [103], which is also observed in the brains of aged mice [102]. Akt activity is upregulated in the aged fruit fly after treatment with growth factor. In contrast, RNAi downregulated Akt phosphorylation and decreased neuronal death, thereby improving starvation conditions and locomotor activity in aged and Aβ42-induced flies [104]. However, the exact molecular mechanisms that associate the origin of AD disease with glucose and insulin metabolic alterations are still unclear.

#### 2.3.2 Glucose Transporters and AD

Glucose transporters or glucose transport proteins (GLUTs) are a family of proteins that facilitate glucose transport into cells [105]. GLUT1 is the predominant transporter responsible for transporting glucose across the blood-brain barrier and into neurons [106]. Studies have shown that abnormalities in glucose metabolism and transporters play critical roles in AD pathogenesis [79]. Additionally, post-mortem studies of AD patients have revealed a decrease in GLUT1 expression in the affected regions of the brain, which proves that impaired glucose transport may contribute to the energy deficits observed in AD [107]. Aβ inhibits the activity of GLUT1, leading to decreased glucose metabolism in neurons [108]. This impaired glucose metabolism can damage neuronal function and contribute to the progression of AD. In addition to GLUT1, other glucose transporters, such as GLUT3 and GLUT4, have also been implicated in AD. GLUT3 acts as the neuron-specific glucose transporter, it is largely observed in the brain, and has been considered as fundamental for neuronal glucose supply. Both GLUT1 and GLUT3 are insulin independent for membrane translocation. The hippocampal GLUT4 protein the same functional role it plays in the classic insulin-sensitive tissues [108]. Research has demonstrated that alterations in the expression and function of these transporters may affect glucose uptake and metabolism in the brain, further exacerbating the energy deficits observed in AD [109].

Although dysregulated glucose metabolism, insulin resistance, altered GLUT expression, and glycation contribute to the development and progression of AD, future research is required to elucidate the precise mechanisms and develop effective therapeutic interventions. Nonetheless, targeting glucose metabolism has great potential to reduce the impact of AD and improve the quality of life of affected individuals. The relationship between glucose dysregulation and AD emphasizes the importance of maintaining healthy blood glucose levels to maintain brain health. Moreover, individuals can manage the risk of developing AD by adopting a healthy lifestyle, including regular exercise, a balanced diet, and proper management of chronic conditions such as diabetes. However, these findings provide valuable insights into potential strategies for prevention and treatment. Further studies are needed to fully understand the complex relationship between glucose dysregulation and AD.

### 2.4 Glucose Dysregulation in Depression

**Fig. (4):**
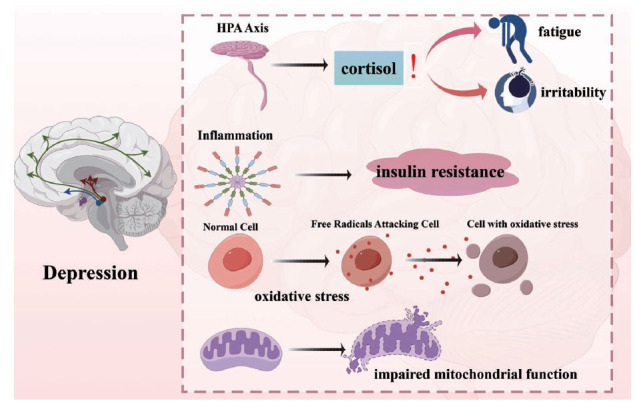
Depression is associated with several physiological changes that affect the glucose metabolism. Multiple mechanisms have been proposed to explain the relationship between glucose metabolism and depression, including the dysregulation of the HPA axis, inflammation-induced insulin resistance, oxidative stress, and impaired mitochondrial function.

Depression is associated with several physiological changes that affect the glucose metabolism [110]. Based on the study, individuals with depression had reduced glucose metabolism in certain regions, particularly the prefrontal cortex and hippocampus of the brain [111]. Further, it has been proved that this decrease in glucose metabolism may lead to impaired brain function and contribute to depressive symptoms [112]. Another study revealed that individuals with depression have higher levels of insulin resistance, which leads to elevated blood glucose levels [113]. Insulin resistance is linked to inflammation and oxidative stress, and may disrupt glucose metabolism in the brain, leading to mood and behavioral changes [114]. Moreover, multiple mechanisms have been proposed to explain the relationship between glucose metabolism and depression, including the dysregulation of the HPA axis, inflammation-induced insulin resistance, oxidative stress, and impaired mitochondrial function (Fig. **4**) [115, 116]. These processes can disrupt neuronal homeostasis, leading to depressive symptoms.

The HPA axis is a complex communication system between the hypothalamus, pituitary gland, and adrenal glands that regulates the body’s response to stress [117]. Dysregulation of the HPA axis results in abnormal cortisol levels and depression by contributing to its symptoms, such as fatigue, irritability, and difficulty concentrating [118, 119]. Additionally, studies have shown that individuals with depression tend to have higher cortisol levels [120]. Prolonged psychological stress has been associated with adverse effects on HPA axis function, increasing the risk of depression [121]. Studies have shown that prolonged exposure to high cortisol levels can damage the hippocampus, a brain region that is important for memory and emotional regulation [122]. This damage can further increase symptoms of depression, making it more difficult for individuals to cope with stress, this hyperactivity depression disorder can increase cortisol levels, a hormone that can impair insulin sensitivity and lead to glucose dysregulation [123]. Elevated levels of glucocorticoid (GCs) over an extended time are associated with neuronal damage and apoptosis and have been observed in individuals with AD and depression [124]. Moreover, the treatment of depression often involves a combination therapy, medication, and lifestyle changes.

### 2.5 Oxidative Stress and Neuroinflammation in the Pathogenesis of AD and Depression

Mitochondria, the powerhouses of cells responsible for producing energy, and disruptions in their function have been observed in individuals with AD [125]. Therefore, mitochondrial function, oxidative stress, and inflammation may play a role in the association between glucose metabolism and AD. Mitochondrial dysfunction is a pathological link between blood glucose and AD, which can reduce mitochondrial respiration and decrease energy synthesis, causing synaptic injury and AD development in diabetic mice [126]. Further studies have revealed that Aβ leads to mitochondrial dysfunction and induces metabolic disorders, including the tricarboxylic acid (TCA) cycle [127]. Next, mitochondrial dysfunction is associated with oxidative stress and inflammation. Oxidative stress occurs when an imbalance between free radicals and antioxidants is implicated in AD development [128]. They can also cause mitochondrial dysfunction and neuronal death. Inflammation, which is the body’s response to injury or infection, plays a role in AD progression [129]. Reactive oxygen species (ROS) are molecules produced during normal cellular metabolism that can damage cellular components such as proteins, lipids, and DNA, leading to dysfunction and, ultimately, cell death [130]. Although ROS play essential roles in various physiological processes, excessive ROS production can cause oxidative stress, which has been implicated in the pathogenesis of several neurodegenerative diseases, including AD [131]. In the brain, oxidative stress contributes to the formation of beta-amyloid plaques and neurofibrillary tangles, which are characteristic features of AD [132]. Additionally, ROS have been shown to activate inflammatory pathways and disrupt synaptic function, further exacerbating neurodegenerative processes [133]. In support of this, several studies have evidenced the increased levels of ROS and markers of oxidative damage in the brains of AD patients [134]. Furthermore, genetic and environmental factors that promote ROS generation or impair antioxidant defense mechanisms are associated with disease development [135]. For instance, mutations in genes encoding antioxidant enzymes, such as superoxide dismutase and glutathione peroxidase, are linked to an increased risk of AD [136]. Since oxidative stress plays a significant role in AD, targeting ROS and restoring redox balance has emerged as a potential therapeutic strategy. Antioxidants, such as vitamin E, vitamin C, and polyphenols, reduce oxidative damage and improve cognitive function in AD [137]. However, clinical trial outcomes investigating the efficacy of antioxidant therapy in humans have been inconsistent, emphasizing the challenges of targeting oxidative stress in AD patients.

It has been evidenced from studies that individuals with depression have higher levels of ROS and lower levels of antioxidants [138], which neutralize ROS and prevent oxidative damage [139]. An imbalance between ROS and antioxidant levels can lead to oxidative stress [140]. Furthermore, oxidative stress influences various neurotransmitter systems in the brain, including serotonin, and may disrupt normal function and lead to inflammation in the brain [141]. In addition, several studies have demonstrated the effectiveness of antioxidants, either alone or in combination with traditional antidepressants, in reducing oxidative stress and improving the symptoms of depression [142]. Although targeting oxidative stress with antioxidant therapy may lead to the development of novel and more effective treatments for depression, further research is required to fully understand the role of ROS in depression. In conclusion, insights into oxidative stress in depression may help researchers develop novel therapeutic strategies by targeting this underlying mechanism and improving outcomes for individuals with depression.

Neuroinflammation is a significant factor in the development of depression [13]; patients exhibit increased plasma levels of proinflammatory cytokines (IL-1, IL-6, IL-8, IL-12, IL-1β, and interferon-γ) and decreased levels of anti-inflammatory cytokines (IL-4, IL-10, and TGF-β1) [143]. As this suggests a relationship between inflammation and depression, the exact nature of this association remains under investigation. Chronic inflammation can affect the balance of neurotransmitters in the brain, such as serotonin and dopamine, which are crucial for mood regulation [144]. Inflammation may also impact the brain structure and function, particularly in regions involved in emotional processing and stress responses [145]. Brain areas important to emotional regulation have been shown to be directly influenced by excess activation of brain cytokine networks [146]. Microglia is the primary inflammatory cell type in the brain, it can produce proinflammatory cytokines [147]. Brain regions such as the hippocampus, amygdala, and anterior cingulate cortex have been reported to particularly be influenced by increased cytokine levels, which are areas that have been repeatedly associated with depression [148]. Inflammation can lead to oxidative stress, an imbalance between free radicals and antioxidants in the body, and cause damage to tissues and organs, resulting in many diseases [149]. Oxidative stress plays a pathogenic role in chronic inflammatory diseases, antioxidant defenses are diminished and oxidative stress is elevated in depression.

Inflammation is a crucial pathophysiological link between major depression and metabolic syndromes, including glucose levels [150]. Higher concentrations of chemokines and IL-6 have also been observed in patients with depression [151]. Proinflammatory cytokines may cause a decrease in serotonin levels, as well as neurogenesis and synaptic plasticity-physiological states linked to depression [152]. Thus, it can interact with virtually all pathophysiological changes that characterize major depression, thereby influencing neurotransmitter function, synaptic plasticity, and neuronal structure. As a result of immune activation, changes in the tryptophan-kynurenine pathway play a significant role in dysfunctional neurotransmitter systems in the brain and contribute to changes in the brain structure and function that characterize depression [153]. Therefore, the neuroinflammation caused by the accumulation of β-amyloid, the dysregulated activity of microglia and the loss of neuroprotective functions emphasizes the central role of inflammatory processes in the pathophysiology of depression and dementia [154]. Consequently, there is synaptic dysfunction and extensive neuronal damage influencing regions that are crucial for cognitive and emotional control. This series of events highlights potential targets for therapeutic interventions that aim to regulate neuroinflammatory pathways in the context of depressive and neurodegenerative disorders.

## 3 DEPRESSION AS A RISK FACTOR FOR AD

Depression is a potential risk factor for AD [155]. Some studies have shown that individuals with depression are more likely to develop AD later in their life [156]. Further, depression is present in approximately 50% of patients with AD. Various potential mechanisms have been proposed to explain the connection between depression and AD [156], including neuroinflammation, oxidative stress, dysregulation of the HPA axis, and changes in the neurotransmitter systems [157-159]. Next, changes in cortisol levels and hippocampal atrophy due to depression may further increase the risk of AD [160] Disruptions in glutamatergic synaptic signaling and decreased BDNF levels are also contributing factor [161]. For instance, chronic stress and inflammation associated with depression can damage brain cells and increase the production of Aβ plaques [162]. Moreover, depression can alter the structure and function of the brain by reducing the hippocampal volume and impairing neurotransmitter function [163]. By the way, methylglyoxal (MG) acts as the consequences of alterations in glucose regulation and its association with depression and AD. For example, MG is increased in T2DM and may be a significant mediator of the overlaps of major depressive disorder (MDD) with AD [164, 165]. MG can arise in any cell, typically as a consequence of hyperglycemia. MG is associated with stress-induced MDD with cognitive deficits, as seen in preclinical models as well as being an AD risk factor [166, 167]. The damaging effects of MG are typically modelled as being mediated by its role as a precursor for the production of advanced glycation end products (AGE), which activate the receptor for AGEs (RAGE). RAGE activation is intimately linked to stress/ depression and AD [168, 169]. As well as T2DM, MG is increased exclusively in astrocytes in the CNS, as shown in the spontaneously hypertensive rodent model [170], suggesting that MG may be an important coordinator of wider metabolic syndrome and the association of metabolic syndrome with a diverse array of medical conditions, including MDD and AD [171, 172]. Recent work has highlighted the importance of astrocytes in the association of MDD with AD [173].

## CONCLUSION

**Fig. (5):**
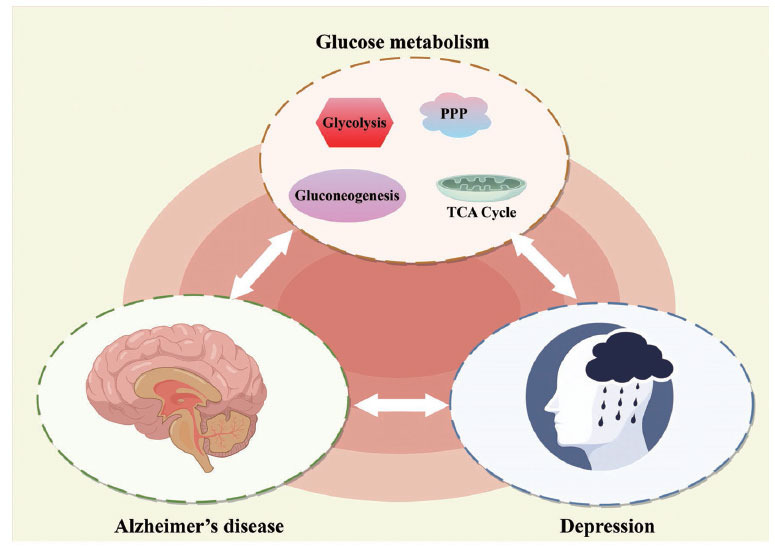
Glucose metabolic abnormalities to depression and AD. The relationship between depression, AD, and abnormalities in glucose metabolism suggests a complex interaction between these conditions. Understanding the intricate relationships between these disorders can lead to the development of innovative therapeutic strategies that target metabolic irregularities.

Understanding the relationship between glucose metabolic abnormalities, depression, and AD offers new treatment possibilities. Targeting glucose metabolism through lifestyle changes, medications, or innovative therapies may be a promising approach for simultaneously managing both conditions. Abnormalities in glucose metabolism play a significant role in the association between depression and AD. The shared pathophysiological mechanisms related to disrupted glucose metabolism contribute to the onset and progression of both disorders. Future research must uncover the underlying molecular pathways and identify potential therapeutic targets for effective management and prevention of debilitating conditions. Glucose metabolism is a critical process that provides energy for cellular functions, and its disruption can lead to various health conditions. Further, recent studies have linked glucose metabolic abnormalities to neurological disorders such as depression and AD. Additionally, both conditions have been linked to glucose metabolic abnormalities, suggesting potential crosstalk between the two diseases. Also, the relationship between depression, AD, and abnormalities in glucose metabolism suggests a complex interaction between these conditions. Understanding the intricate relationships between these disorders can lead to the development of innovative therapeutic strategies that target metabolic irregularities (Fig. **5**). Hence, future research should focus on uncovering the molecular mechanisms that drive this interaction to better understand these conditions and identify potential therapeutic targets.

## LIST OF ABBREVIATIONS

AD: Alzheimer’s Disease
HPA: Hypothalamic-pituitary-adrenal
T2DM: Diabetes Mellitus Type 2
Aβ: Amyloid-beta
NFTs: Neurofibrillary Tangles
ROS: Reactive Oxygen Species
APP: Amyloid Precursor Protein
PSEN1: Presenilin 1
PSEN2: Presenilin 2
APOE: Apolipoprotein E
CSF: Cerebrospinal Fluid
SSRIs: Selective Serotonin Reuptake Inhibitors
5-HT: 5-hydroxytryptamine
CORT: Corticosterone
BDNF: Brain-derived Neurotrophic Factor
GABA: Gamma-aminobutyric Acid
PET: Positron Emission Tomography
PPP: Pentose Phosphate Pathway
HK: Hexokinase
PFK: Phosphofructokinase
G6PD: Glucose-6-phosphate Dehydrogenase
IRS1/2: Insulin Receptor
MAPK: Mitogen-activated Protein Kinase
GSK3β: Glycogen Synthase Kinase 3β
PI3K: Phosphatidylinositol 3-kinase
PIP3: Phosphatidylinositol 3,4,5-trisphosphate
CNS: Central Nervous System
BBB: Blood-brain Barrier
IR: Insulin Resistance
IGF1: Insulin-like Growth Factor 1
IRS-1 p^S616^: IRS-phosphorylated at serine 616
TCA: Tricarboxylic Acid
GLUTs: Glucose Transport Proteins
GCs: Glucocorticoid
MG: Methylglyoxal
MDD: Major Depressive Disorder
AGE: Advanced Glycation End Products
RAGE: Activate the Receptor for AGEs
